# Spontaneous Collapse Theories and Temporal Primitivism about Time’s Direction

**DOI:** 10.1007/s10701-022-00632-3

**Published:** 2022-10-06

**Authors:** Cristian López

**Affiliations:** Université de Lausanne, Lausanne, Switzerland

**Keywords:** Time’s direction, Collapse theories, Primitivism, Time-reversal invariance, Quantum mechanics

## Abstract

Two views on the direction of time﻿ can be distinguished—primitivism and non-primitivism. According to the former, time’s direction is an in-built, fundamental property of the physical world. According to the latter, time’s direction is a derivative property of a fundamentally directionless reality. In the literature, non-primitivism has been widely supported since most (if not all) our fundamental dynamical laws are time-reversal invariant. In this paper, I offer a way out to the primitivist. I argue that we do have good grounds to support a primitive direction of time in the quantum realm. The rationale depends on exploiting the metaphysical and dynamical underdetermination of quantum theories to make a case in favor of primitivism. In particular, primitivism can be grounded in spontaneous collapse theories (e.g., GRW and CSL). The specific sense in which these theories capture a primitive direction of time is that, when the ontology of the theory is seriously taken into account, it does not remain invariant under time reversal. In taking GRW with a matter-density field (GRWm), I will argue that primitivism about the direction of time can be defended in the quantum case.

## Introduction

In the debate about the direction of time in physics, some think that the direction of time is primitive, others that it is derivative or emergent. Whereas primitivists believe that the direction of time is an irreducible feature of the natural world, non-primitivists believe that it is a derivate property, reducible to, or dependent on, an atemporal basis. In the philosophy of physics, non-primitivism has widely been common currency. One of the arguments comes from the concept of time-reversal invariance. In so far as the fundamental laws of physics are believed to be time-reversal invariant, a primitive direction of time looks, at best, unwarranted. This has been generally interpreted in the following way—so far as fundamental physics is concerned, to equip physical theories with a primitive directionality is to equip them with unneeded excessive structure that it is nowhere found in a time-reversal invariant physics. Since it would be an epistemic vice to equip physical theories with unneeded structure, any form of primitivism about the direction of time must be rejected.

One of the consequences of this argument is not only that the direction of time is derivative or emergent, but that its nature must be explained by non-dynamical features, as asymmetric boundary conditions. This informal argument is pervasive in physics. In classical mechanics, in either of its formulations, the fundamental dynamical equations are time-reversal invariant. Then, from the perspective of classical physics, nothing in the classical dynamics entitles us to equip the physics with a primitive direction of time (See [25, 26 for criticisms]). Any temporal asymmetry will then be found, for instance, in the boundary conditions. In quantum theories, the argument is similar. In non-relativistic quantum mechanics, physical states are given by quantum states that evolve unitarily, linearly, and deterministically by the Schrödinger equation. It happens that the Schrödinger equation is time-reversal invariant. Therefore, nothing in the quantum dynamics entitles us to equip quantum physics with a primitive directionality (see [1, 9, 30] for criticisms). Once again, any explanation of the manifested temporal asymmetry should come from non-dynamical elements.

These arguments based on time-reversal invariance have typically left primitivism in an uneasy situation—why the direction of time can be said to be primitive despite having, all the way down, time-reversal invariant dynamics. In general, this has led to deflate the relevance of arguments based on time-reversal invariance in discussing the direction of time in physics (see [1, 33, 59], or to defend temporal primitivism on different grounds (see [35]. In this article, my aim is to resist the main non-primitivist argument based on time-reversal invariance, offering a way-out to primitivism about the direction of time. However, I will not reject the importance of time-reversal invariance for the discussion as others have done, but I will assume that the concept is greatly relevant. My argument rather focuses on a distinction between *weak* and *strong* time-reversal invariance. Whereas the former depends on preserving the space of solutions of dynamical equations, the latter on also preserving the ontological content of the theory. I will then argue that in non-relativistic quantum mechanics temporal primitivism can be defended by adopting spontaneous collapse theories, in particular, GRW-models with a matter-density field (GRWm). Though these theories are weakly time-reversal invariant, they violate strong time-reversal invariance. Therefore, temporal primitivism can be suitably grounded in GRWm.

## Primitivism and Time-Reversal Invariance

### Primitivism vs. Non-primitivism

In the philosophy of physics literature, the direction of time has typically been accounted for in terms of the thermodynamic asymmetry. The fact that entropy tends almost always to increase, but not to decrease plus some time-asymmetric boundary conditions were thought to be the foundations of the manifested directionality of time [1, 8, 25, 49]. However, this way to pose the problem of the direction of time may be a bit narrow. The coordination of the direction of time with the thermodynamic asymmetry is just one way (a strongly reductionist way) to explain why the world looks so time asymmetric, but there are alternatives to explain (or capture) the direction of time. Hence, a more general way to pose the problem, to comprehend other alternatives, seems to be needed.

The problem of the direction of time in its most general way can be formulated in terms of whether the world comes in *some way* equipped with a temporal asymmetry that allows us to *substantially* distinguish between the past-to-future from the future-to-past direction. In the metaphysical parlance, three theories of time, connected with three structures of time, can be distinguished: A-theories, B-theories, and C-theories. The terminology was proposed by McTaggart [37] as alternative ways to consider time and change. Whereas the relevant distinction between the A- and B-structures is that the former implies a temporal dynamics and the latter does not, the difference between the B- and the C-structures is that C-structures lack a direction (see [17]*.* To put it differently, whereas B-structures determine an order and a direction, C-structures only determine an order among events.^1^

So, one way to reformulate the problem of the direction of time more precisely is whether the (natural) world comes equipped with, at least, a B-structure, that is, with a temporal structure that allows univocally arranging the events in “…before… later” relations. If it does not, then events can only be ordered in terms of a relation of “betweenness”, exhibiting a C-structure. Intuitively, the world around us (or, better, the distribution of matter and energy of our universe) looks manifestly time asymmetric, which suggests that it instantiates at least a B-structure. Yet, it might be the case that the world *looks* temporally asymmetric, but it is not fundamentally so—it might be the case that the experienced temporal asymmetry comes out from a more fundamental directionless basis. Hence, the problem of the direction of time gets an interesting twist. The world might *seem* to be equipped with, at least, a B-structure, but it might * fundamentally* be equipped with a C-structure. So, the problem of the direction of time boils down to whether the direction of time is fundamental or not.

There are two general answers to this problem. **Primitivism** about the direction of time (or “temporal primitivism”) holds that the world comes *fundamentally* equipped with, at least, a B-structure that is irreducible to an underlying directionless basis. Then, the manifested temporal asymmetry (or any temporal predicate) supervenes upon the primitive temporal directionality. There would be many ways to characterize this view, but one of them is in terms of “dynamical temporal primitivism”—something inherent (or intrinsic) to the fundamental dynamics of the world differentiates between both temporal directions. A common way to see whether dynamical primitivism about the direction of time holds is through the concept of time-reversal invariance (TRI from now on) —If the fundamental dynamics of the world turns out non-TRI, then it would be a (strong) sign of an inbuilt, primitive temporal directionality (see [24]. However, temporal primitivism does not need to be identified with dynamical temporal primitivism. In different versions, temporal primitivism can be related to the structure of space–time (see, for instance, Tim Maudlin’s view on time [35, 36] John Earman’s Time Direction Heresy [13], Olimpia Lombardi and Mario Castagnino’s global, geometrical approach 2009, and Cristian Lopez and Michael Esfeld’s relational primitivism 2022). None of these views rely on the concept of (non)-TRI, but they do make a case for temporal primitivism. In the rest of the paper, I will exclusively focus on temporal primitivism in its dynamical version, dynamical temporal primitivism.

- **Temporal Primitivism** The direction of time is an irreducible, inbuilt feature of the natural world, upon which any other temporal asymmetry (or temporal predicate) supervenes.
- **Dynamical Temporal Primitivism** A primitive direction of time is manifested in physical theories’ fundamental dynamics, in particular, whether it is TRI or not

How is it supposed to work? Suppose that a law $\mathcal{L}$ rules the transition $A-B-C-D$ with $+t$. If the law is TRI, then the time-reversed law, ${\mathcal{L}}^{T}$ should generate a time-reversed sequence that is dynamically equivalent to the first (that is, it is also a solution of the dynamical equation), ${D}^{T}-{C}^{T}-{B}^{T}-{A}^{T}$. Then, the law $\mathcal{L}$ generates a symmetric twin-pair. But, if the law were non-TRI, then the time-reversed sequence would not be a solution of the dynamical equation. Therefore, non-TRI laws do not generate symmetric twin-pairs, but only one of them. Conceptually, this means that for any sequence in one direction of time that is ruled by a non-TRI dynamical law, there will not be a dynamically equivalent sequence in the opposite direction. Therefore, a substantial distinction between temporal sequences can be drawn on the basis of the structural features of the dynamics. Since dynamical temporal primitivism relies on the structural features of a theory’s dynamics, the main challenge is, then, to give the non-TRI law (see [3, 24], among others).

**Non-Primitivism** rather holds that the manifested temporal asymmetry is derivative. The world, at its fundamental level, comes equipped with just a C-structure, that is, a structure that prescribes an order, but not a temporal direction. At some point, as we go higher in the hierarchy, we start to find B-structured phenomena, but they are non-fundamental—they can be reduced to C-structured phenomena that underly them. Ultimately, everything supervenes upon (or emerges from) a time directionless basis (see [29]. In this way, we can explain any temporal asymmetry as well as any temporal predicate, not by relying on a primitive direction of time, but by explaining how temporally asymmetric phenomena “emerge” from a temporal directionless basis. As in the previous case, there are many instances of temporal non-primitivism. Dynamical temporal non-primitivism holds that if the fundamental dynamics of the world is TRI (or “frozen”, as some speculations in quantum gravity submit), then any temporal asymmetry cannot be grounded in the fundamental dynamics but must be explained by non-dynamical elements. The challenge here is to tell a story that satisfactorily connects the manifested temporal asymmetry with the temporally symmetric basis. For instance, the existence of TRI laws plus the postulation of special initial conditions at the beginning of the universe (i.e., the Past Hypothesis, see [1, 48] renders the direction of time non-fundamental, but it allows explaining the thermodynamic asymmetry as well as any other asymmetry (e.g., the epistemic arrow of time).

### Time-Reversal Invariance: Weak or Strong?

From what has been said so far, it follows that one of the main arguments against dynamical temporal primitivism is that all our fundamental physical laws seem to be TRI. This would strongly favor a C-theory of time since nothing in the fundamental dynamics of the world seems to distinguish between the past and the future directions. This claim has appeared in many places throughout the relevant literature (see, for instance, [13, p. 23, 24, p. 39, 55, pp. 6, 12, 47, p. 116, 35, p. 266, 59, pp. 262–263]. The incompatibility between having TRI laws in our best physical theories (i.e., in classical statistical mechanics, relativistic and non-relativistic quantum mechanics, etc.), on the one hand, and adopting temporal primitivism on the other, has been a distinctive mark of the literature.

The general argument can be sketched as follows.

P1: If the fundamental dynamics of our best physical theories is TRI, then temporal primitivism is unwarranted (see, for instance, [24].^2^
P2: It happens that the fundamental dynamics of our best physical theories is TRI
C: Therefore, temporal primitivism is unwarranted.

Farr [18] correctly notes that this argument is similar to the one of Russell [52]: whereas Russell relies on TRI to make a case against causation as an ontological feature, the argument employed here relies on TRI to make a case against temporal primitivism. But the structure is to a good extent the same. In any case, the truth conditions of the premises of this argument greatly hinge upon the concept of TRI.

As explained in the previous section, TRI means, prima facie, that if a physical process is in accordance with a dynamical law, then the time-reversed process is also in accordance with the law. More precisely, TRI preserves the space of solutions of dynamical equations by mapping solutions into solutions, and non-solutions into non-solutions, keeping the dynamical equation invariant. More formally, time reversal is a symmetry operator that acts upon mathematical structures (generally, differential dynamical equations) preserving some formal relations that conserve the space of solutions. A formal definition (**FD**) below.

**FD** Let $L$ be a law represented by an equation$E(s, {O}_{i}, {D}_{j}, x, t)=0$, where $s$ represents a state, the ${O}_{i}$ represent observables, the ${D}_{j}$ represent differential operators, *x* the position and *t* the time coordinate as independent parameters. Let $T$ be an operator acting upon the objects appearing in the equation, such that $T$ reverses the time coordinate,$T:t\to -t$; it keeps the positions unchanged,$T:x\to x$; and it transforms the observable and states in such a way to generate a backward evolution,$T:s\to \widetilde{s} , T:{O}_{i}\to \widetilde{{O}_{i}}$. If the space of solutions of $L$ is preserved and $L$ remains invariant under *T*, then it is said to be TRI.

**FD** is a broad definition. In general, it just says that any time-reversal transformation generates an inversion of a state sequence, $T:\left[A-B-C-D\right]\to [D-C-B-A],$ and also the inversion of each state, $T: S\to {S}^{T}$ (for more details, see [55, 57]). However, the implementation of time reversal is to be theory-relative, since it will depend on the nature of the states and of the observables how they should transform within a theory. In Hamiltonian classical mechanics, for instance, time reversal involves a transformation $T$ that reparametrizes the time coordinate, changes the sign of the ${p}_{i}$ s, and leaves ${q}_{i}$ s unchanged (with the ${p}_{i}$ and the ${q}_{i}$ referring to conjugate momentum and generalized position, respectively), transforming the set of all smooth curves $(q\left(t\right), p\left(t\right))$ through phase space. This transformation is directly related to a symmetry property of the Hamiltonian, ${H(q}_{i}, {p}_{i})={H(q}_{i}, {-p}_{i})$. If a system’s Hamiltonian satisfies this symmetry, then the equations of motion are TRI under $T$, conforming with **FD**.

We can wonder whether **FD** is too weak, mainly if the concept is to be used in a philosophical argument. Intuitively, it is blatant that two evolutions related by time reversal according to **FD** may well be solutions of the dynamical equations but be nonetheless inequivalent in many respects. Gordon Belot [5], in an influential article about symmetries and equivalence, claims that if a definition of symmetry (like **FD**) is not complemented with some epistemic, modal, or ontological content, it is a “recipe for a disaster”, since it will render models as equivalent that obviously are not in some important aspects.^3^ In virtue of this, it might be helpful to distinguish two ways to understand TRI–*weak* and *strong*.

*Weak* TRI is fully given by **FD**. Though most physical theories are weakly TRI (or can easily be made TRI by defining the right time-reversal transformation) the definition is not trivial. For instance, suppose that our world is such that its fundamental dynamical equations are those given by Newtonian classical mechanics, but we discover a fundamental (non-conservative) drag force. Newton’s Second Law, then, should include such a force in its most basic expression (i.e., the free-particle model plus the drag force). So, for any classical evolution, the system will tend to slow down if it moves against the drag force. It is easy to see that classical evolutions will be non-TRI, since their time-reversed counterpart will exhibit a system spontaneously accelerating from relative rest, without exerting any force upon it. Time reversal, in this case, transforms solutions into non-solutions. Therefore, it violates weak TRI.

**Weak TRI** A dynamical law (or set of laws) is weakly TRI if the time-reversal transformation only preserves the space of solutions (**FD**)

*Strong* TRI is far more interesting. It of course includes **FD**, but it extends the preservation of further content. For instance, we could demand a symmetry transformation, where time reversal is a particular case, to preserve the *observational* content (see, for instance, [11, 27]. A violation of strong TRI will then amount to a difference in the observational content between past-headed and future-headed evolutions. But if the observational content remains the same, then strong TRI is preserved. A straightforward example is Galilean boost in Newtonian classical mechanics and the consequent elimination of absolute velocities—since boosting a classical system does not affect the observational content and velocities are rendered relative to another system, then absolute velocities are not observed. Therefore, they plausibly do not exist. Analogously, a primitive direction of time can be discarded if there is no change in the observational content when time reversal is applied.^4^

Another way to complement **FD** is by requiring that the *ontological* content of the theory be preserved (see [2]. The ontological content of a physical theory is given both by its basic (or primitive) ontology (the “building blocks”) and its derivative (or secondary) ontology (entities, properties or relations that supervene upon the building blocks). It is the latter that connects with the observational content of the theory and supervenes upon the former. A violation of strong TRI would for instance entail that the primitive ontology is not preserved —some relations, properties or entities are affected by reversing the direction of time. In the rest of the paper, I will use this characterization of strong TRI.^5^

**Strong TRI** A dynamical law (or set of laws) is strongly TRI if the time-reversal transformation preserves the space of solutions (**FD**) and the *ontological content* of the theory as well.

In the literature on the direction of time in physics, the distinction between weak and strong versions of TRI has not been so clear, resulting in some ambiguities. According to my view, that a theory be weakly TRI means that it can work equivalently well both with $+t$ and $-t$. A violation of weak TRI means, by definition, that a dynamical law will only work either $+t$ or $-t$, but not with both. In other worlds, weakly non-TRI laws will yield solutions in one temporal direction, but not in the other. Yet, this does not mean that if a theory is weakly TRI, it will also be strongly TRI. A theory may violate strong TRI but be weakly TRI: the space of solutions of its dynamical equations is preserved, but the ontological content changes when time is reversed. For instance, suppose that the universe has a certain mass-energy distribution over a span of time. Then, we want to know if the same distribution could be obtained if time had run backward. If the same distribution is obtained, then strong TRI is respected; but if we obtain a different mass-energy distribution, that is nonetheless a solution of the dynamical equations for that universe, then weak TRI still holds, but strong TRI is violated. This inequivalence between ontological content would then be a way to distinguish between the past-to-future and the future-to-past directions of time.

The distinction between weak and strong TRI is valuable for the following reasons. First, if TRI is taken simply in its formal aspects (i.e., weak TRI), it is then a bit unjustified that the mere formal fact that physical theories be weakly TRI is a sufficient reason to draw a substantial conclusion about the direction of time. Second, the very notion of symmetry requires a more substantial interpretation if meant to be useful for philosophical arguments, as pointed out earlier. The idea of complementing the formal definition of TRI with the preservation of the ontological content would not only provide us with a more robust notion of TRI but also avoid many cases in which there may be differences between time-reversed cases that are nonetheless weakly TRI (i.e., cases where solutions are transformed into solutions by time reversal, but the ontological content is not completely preserved).

## The Direction of Time and Quantum Mechanics

As far as we know, the world can be adequately described by quantum theories. In the non-relativistic regime, standard quantum mechanics satisfactorily accounts for phenomena, while in the relativistic one, relativistic quantum field theories do this job. It happens that both standard quantum mechanics and quantum field theories are said to be TRI: the Schrödinger equation as well as the Dirac and Klein-Gordon equations are TRI. Temporal non-primitivists have taken this fact to hold that the direction of time is therefore non-fundamental, but emergent—so far as the non-primitivist is concerned, the direction of time is to be found at higher levels, but it is in no way inherent to the fundamental quantum dynamics. How does the temporal primitivist face the challenge of TRI in the quantum regime? The best strategy, I will argue, is first to draw the attention towards strong TRI, rather than weak; second to use the dynamical and ontological underdetermination of quantum theories in her favour.

### The Anti-primitivist Argument

For simplicity, I will circumscribe the argument to the non-relativistic regime. In standard quantum mechanics, the fundamental dynamics is completely given by the Schrödinger equation. For a single non-relativistic system in one dimension, the equation takes the following form:

1 $$i\hslash \frac{\partial }{\partial t}\psi \left(x,t\right)=\left[-\frac{{\hslash }^{2}}{2m}\frac{{\partial }^{2}}{\partial {x}^{2}}+V\left(x,t\right)\right]\psi \left(x,t\right)$$

where $\psi (x,t)$ is the wave-function (in the position representation), $V(x,t)$ is the potential, $\hslash$ is the reduced Planck constant, $i$ is the imaginary unit, and $m$ the mass of the system. Solutions of the Schrödinger equation are possible evolutions of the wave-function.

If dynamical primitivism can be supported in non-relativistic quantum mechanics, then (Eq. 1) should be either non-weakly or non-strongly TRI. To check this, we need to define a time-reversal operator that implements the idea of time reversing (Eq. 1). Though there has been some discussion about how time reversal must be implemented in standard quantum mechanics (see [9, 31, 50], the common view has it that time reversal is implemented by an anti-unitary operator *T* that transforms the time parameter as $T:t\to -t$, that keeps the position $T:x\to x$, and, interestingly, that transforms the wave-function into its complex conjugate $T:\psi \to {\psi }^{*}$. One of the reasons to implement time reversal in terms of an anti-unitary operator is that it keeps the spectrum of the Hamilonian bounded-from-below, whereas a unitary implementation does not (see [31, 50] for further details and discussion). In this way, we can guarantee that any time-reversed Hamiltonian keeps its spectrum invariant. When applying the anti-unitary *T* upon (Eq. 1), the Schrödinger equation is left invariant—a wave function evolving forward in time is symmetric to its complex conjugate evolving backward.

The anti-Primitivist Argument in the quantum case is just an instance of the more general argument sketched earlier (Sect. 2.2.):

MQ1: The fundamental dynamics of non-relativistic quantum mechanics is given by the Schrödinger equation.
MQ2: If the Schrödinger equation is TRI, then temporal primitivism is unwarranted in non-relativistic quantum mechanics.
MQ3: It is a matter of fact that the Schrödinger equation is TRI.
C: Therefore, temporal primitivism is unwarranted in non-relativistic quantum mechanics.

### The Primitivist Argument: Dynamical and Ontological Underdetermination

The burden is now on temporal primitivism’s side: she has to explain why temporal primitivism is not unwarranted, despite the Schrödinger equation being TRI. A laborious way out is to deflate the meaning of time reversal (see [1, 10, 13, 33]. It can be argued that time-reversal invariance is an epistemic feature of physical theories, from which we should not extract any ontological conclusion (e.g., [33], or that the concept of time reversal is inadequate in relativistic and cosmological contexts [10, 13]. An alternative is to insist that there are actually other fundamental non-TRI laws. The rare decay of K^0^-mesons, discovered by Cronin and Val Fitch in 1964, seems to point towards that direction. However, it is not completely clear how the rare decay of such exotic subatomic particles can serve to temporal primitivism (see [35, 40] for a positive view, see [24, 45, 53] for criticisms). Regardless how viable these alternatives may be, I believe that temporal primitivism can be defended without rebutting the main premises of the anti-Primitivist Argument, nor relying on rare empirical evidence. The strategy will depend on turning the attention towards strong TRI, rather than weak TRI. In doing so, ontology must come in.

If physical theories should strive for some form of understanding of the natural world, then standard quantum mechanics is not a complete theory from that viewpoint. For empirical adequacy, it does it satisfactorily well; but for more substantial issues it delivers an unacceptable picture of what the world is like if the theory is taken to be (approximately) true. One of the reasons is the measurement problem (see [34, 58] for two different formulations), which pushes us to either add further structure to the theory or to modify it. However, there is not a single way to do this, but many. All of them share the empirical content of standard quantum mechanics and, thereby, are (largely) empirically equivalent.^6^ Therefore, what goes by “standard quantum mechanics” suffers not only from *metaphysical* underdetermination (that is, many ways in which the ontology may be like at the quantum level), but also from *dynamical* underdetermination (that is, many ways in which the ontology may behave at the quantum level).^7^

So, defenders of temporal primitivism may argue that MQ3 in the anti-Primitivist Argument is vague. First, does it refer to weak TRI or to strong TRI? It may be the case that a physical theory be weakly TRI but not strongly TRI. Second, it may be argued that we should not only look at the Schrödinger equation, but also to alternative dynamics that seek to overcome the measurement problem. Even though the Schrödinger equation is both weak and strong TRI, additional structure may break TRI in either sense. Be that as it may, these are hopes for temporal primitivism. The argument for primitivism is then in need of two assumptions. First, that we should not look only at standard quantum mechanics, but also at alternative quantum theories (those that do solve the measurement problem). Second, that we must assess both weak and strong TRI. A violation of either would give temporal primitivism some grips on the physics. On this basis, temporal primitivism may use the dynamical and the metaphysical underdetermination of standard quantum mechanics in her favour, choosing the quantum theory that best suits her philosophical views on time.^8^

## Spontaneous Collapse Theories and Temporal Primitivism

There is in fact a family of quantum theories that reproduces the quantum statistics and upon which temporal primitivism may, in principle, have some grip. This family is the *spontaneous collapse theories*. ﻿They postulate that the quantum dynamics is not always unitary and deterministic, but that once in a while it undergoes collapses, or “jumps”, that guarantee definite outcomes. Conforming to a common opinion, spontaneous collapse theories are non-TRI. In this section, I will briefly outline some of the features of the spontaneous collapse theories. For simplicity, I will mainly take the GRW theory, though my view can be extended to others too. My argument has two steps. First, I will show that GRW is weakly TRI, contrary to the common view. This point has been also made by Bedingham and Maroney [6, 7], with whom I partially agree. Second, I will argue that, even though GRW is weakly TRI, it violates strong TRI. To illustrate this, I will focus on GRW with a mass-density field (GRWm). My point is that the ontological content of GRWm is not preserved under time reversal. Therefore, temporal primitivism can be suitably defended if GRWm is adopted.

### Spontaneous Collapse Theories

Spontaneous collapse theories were introduced in the 70 s and 80 s to solve the measurement problem by modifying the standard dynamics for the quantum state [19, 21, 22, 43, 44]. The idea was to substitute ad-hoc older versions of collapse theories that mysteriously invoked the notion of “measurement” or “observer” (e.g., [12, 57] by a single evolution law that spontaneously produces collapses without resorting to classical measurement apparatuses. Even though there have been different spontaneous collapse theories,^9^ the most popular has been GRW [21].

In GRW, quantum systems in isolation *almost* always evolve according to the standard quantum dynamics (the Schrödinger equation), having their wave functions in a superposition of the position operator. Yet, collapses occur spontaneously in function of a mean frequency $\lambda$ (a new constant of nature for the frequency that a “jump” or “hit” occurs.)^10^, which get the wave function multiplied by a narrow three-dimensional Gaussian function of the position operator (a second new constant of nature appears here, the width $\sigma$ of the multiplying Gaussian curve.)^11^. The spontaneous process of localization is given by ${L}_{x}^{i}$ (the collapse operator) a norm-reducing, positive, self-adjoint, linear operator in the *n*-particle Hilbert space, representing the localization of particle *i* around the point *x* (Ghirardi 1995, p. 174). Then, any spontaneous process is a localization described by

2 $$\left|\psi \right.\rangle \to \left|{\psi }_{x}^{i}\right.\rangle =\frac{\left|{\varphi }_{x}^{i}\right.\rangle }{\Vert {\varphi }_{x}^{i}\Vert }$$

3 $$\left|{\varphi }_{x}^{i}\right.\rangle ={L}_{x}^{i}\left|\psi \right.\rangle$$

Consider the initial wave function of a quantum system *I* at ${t}_{0}$, $\left|{\psi }_{{t}_{0}}^{i}\right.\rangle$. From ${t}_{0}$ to ${t}_{1}$ (where ${t}_{1}={t}_{0}+\Delta {t}_{1}$) the wave function will evolve according to the standard quantum evolution. It is worth noticing that $\Delta {t}_{1}$ is a random time distributed conforming with the exponential distribution with rate $N\lambda$, where *N* is the number of quantum systems. Now, let us assume that at time ${t}_{1}$, the single system *i* suffers an instantaneous collapse with random center *X*. If $\left|{\psi }_{{t}_{0}}^{i}\right.\rangle$ was in a superposition, one of the terms gets multiplied by a finite number, and all the rest by 0. Thereby, all the terms (except that multiplied by a finite number) vanish and the system gets a precise localization centered at, say, ${x}_{k}$. The probability that the *k*th term gets multiplied by a finite number is thus equal to ${\left|{x}_{k}\right|}^{2}$. Then, the algorithm is iterated: the quantum system evolves again according to the Schrödinger equation up to a time ${t}_{2}={t}_{1}+\Delta {t}_{2}$ where a new collapse occurs, and so on.

But which is the dynamics that account for the whole process? The fundamental dynamical equation in GRW is a linear stochastic evolution for the quantum state (see [19].^12^ In the Stranovich form, the dynamical equation is

4 $$\frac{d{\psi }_{w}(x,t)}{dt}=\left[-\frac{i}{h}H+\sum_{i}{A}_{i}{w}_{i}\left(t\right)-\gamma \sum_{i}{A}_{i}^{2}\right]{\psi }_{w}(x,t)$$

It is easy to see that (Eq. 4) looks very much like the standard Schrödinger equation, except for two terms on the right side, where the quantities ${A}_{i}$ are commuting self-adjoint operators and the quantities ${w}_{i}\left(t\right)$ are c-numbers (i.e., classical numbers, not quantum operators).

The single stochastic dynamics then accounts for both micro and macroprocesses. The collapse mechanism merges with the standard quantum evolution. It is noteworthy that the relevant question now is no longer whether the Schrödinger equation is weakly/strongly TRI, but whether (Eq. 4) is.

### Time Reversal, Symmetry, and Ontology

It is common opinion that GRW is deeply non-TRI. This would on its own favour temporal primitivism. Jill North [40], for instance, says:

Not only is wavefunction collapse governed by a fundamental, indeterministic law on this theory (GRW), but by a fundamental, non-time-reversal invariant law. GRW assigns probabilities to the different possible future wavefunctions that a system’s current wave-function could collapse into. [40], pp. 333)

A few lines below,

The theory doesn’t assign probabilities to different possible past wave-functions, given a system’s current wave-function. The collapse law doesn’t say anything about the chances of different past wave-functions. (Ibidem)

Finally,

GRW then says that different things can happen in either direction of time: wavefunctions can collapse in accord with lawful probabilities to the future, not the past. (Ibidem)

Frank Arntzenius [3], in the same line, claims:

Thus, if one believes in some “orthodox” collapse version of quantum mechanics, or one of the more recent Pearle-GRW type collapse, one has reason to believe that time has an objective direction. ([3], p. S218)

Later, he adds.

The conclusion, therefore, is that whether one should believe that time has an arrow depends on one’s interpretation of quantum mechanics: If one adheres to a theory of probabilistic collapse, one has reason to believe that time has an arrow. ([3], p. S222)

Michael Esfeld and Christian Sachse have also argued that GRW is the right sort of theory to explain the origin of the directionality of time:

This interpretation is in the position to explain the origin of the direction of time. Amending the Schrödinger equation with a stochastic term as GRW does in order to account for state reductions has not only the consequence that the dynamics is indeterministic, but also that it is not time-reversal invariant. In other words, the GRW equation is a candidate for a fundamental *law of nature that is not time-reversal invariant*. (Esfeld and Sachs [16], p. 60)

Craig Callender [9] holds the same view as well. He puts it as following:

On collapse theories, a certain feature of the system (e.g., particle number, mass, being observed) will trigger a non-unitary, indeterministic transition from $\psi$ to one of its components ${\psi }_{i}$. This collapse is not governed by the Schrödinger evolution. And in general, there is no way of evolving from the collapsed system back to the uncollapsed system with the same chance. According to collapse theories, there is a preferred orientation to time. (2000, p. 261).

All these quotes aim towards the same direction—collapse theories, and *spontaneous* collapse theories in particular, bear the right sort of dynamics to ground a primitive direction of time.^13^ However, two points are noteworthy. First, they qualify the conclusion differently. For instance, Callender says that in GRW there is no way of evolving from collapsed system back to the uncollapsed system with the “same chance”, whereas North claims that GRW “says nothing” about past-headed wave-functions, or that we cannot assign probabilities to them. Yet, she also says that “different things can happen” in different temporal directions. All these statements are clearly inequivalent— “to say different things” is not the same as “to say nothing”. Second, claims about TRI in these quotes do not distinguish between the weak and the strong versions. So, we need some clarification on all these points.

The GRW dynamics is given by, for instance, the stochastic dynamical law (Eq. 4): any isolated quantum system will almost always evolve unitarily and deterministically, until it undergoes a spontaneous collapse. With this in mind, imagine a one-particle universe. The quantum system’s initial state is in a superposition of the position operator, $\left|{\psi }_{0}\right.\rangle =\sum_{i}{x}_{i}|{x}_{i}\rangle$. This means that the system’s position is “smeared out”. The dynamics predicts the following: the quantum system is to evolve, *towards the future*, unitarily, deterministically and linearly according to the Schrödinger equation for quite a while. But, after enough time has lapsed (say, ${10}^{9}$ years), the stochastic dynamics also tells us that the quantum system is to undergo a collapse, which will localize the system in the physical space, $\left|{\psi }_{n}\right.\rangle =x|{x}_{k}\rangle$. This process is, of course, a solution of the spontaneous collapse theories. Does GRW violate both senses of TRI? Or just only one? Let us start with weak TRI.

Is the GRW dynamics weakly TRI? More precisely, is the time-reversed wave-function also a solution of the GRW dynamics? If we think carefully what it means to time reverse a stochastic dynamics like (Eq. 4), we will note that it cannot mean to reverse the spontaneous localization mechanism in itself, as if there were a way “to uncollapse” the system. Since the dynamics is unique, there is nothing like the spontaneous localization mechanism to be reversed independently from the whole dynamics. What must be reversed is (Eq. 4) as a whole. This means that the time-reversed wave-function cannot mean a “spontaneous delocalization” process, but a “spontaneous localization” process in the opposite direction of time. To be more precise, a quantum state evolving backward in time is to be the conjugate wave-function ${\psi }^{*}(x, -t)$, which will evolve unitarily and deterministically *towards the past* according to a time-reversed stochastic dynamics. But it is also a conjugate wave-function that, after enough time, is to undergo a collapse *in the past*.

It is easy to see that the wave-function evolving forward and the complex conjugate wave-function evolving backward are equally solutions of the GRW dynamics—nothing in the theory tells us that localization processes only occur either in the future or in the past, but that we should expect localization processes to occur *both* in the future and in the past. Neither does the GRW dynamics predict that the probability of undergoing a spontaneous localization is to be different depending on the chosen temporal direction. Thus, time reversal does preserve the space of solutions of spontaneous collapse theories. In this sense, for instance GRW does tell us something about probabilities of wave-functions evolving backward in time (pace North). Actually, it tells us exactly the same thing (pace Callender)—any quantum system will almost always evolve unitarily and deterministically for quite a while, but it is to undergo, both in the past and in the future, a spontaneous localization collapse after enough time. Therefore, the GRW dynamics (and the spontaneous collapse dynamics in general) is weakly TRI, since both preserve the space of solutions and preserve the same algorithm to calculate probabilities.

A similar point was made by Bedingham and Maroney [6, 7]. They claim that spontaneous collapse models can be cast into a time-reversal symmetric formulation under two assumptions: (a) collapse outcomes are the primitive objects of the theory, and (b) the wave-function just encodes the past history of collapses (and, thus, is just epistemic). According to them, given a “set of collapses there are two equivalent pictures of a collapsing wave function: one going forward in time and one going backward in time, each satisfying the dynamical rules” ([7], p. 694). The time-reversal symmetry appears because the outcomes of subsequent collapses satisfy a probability rule of the same form ([7], p. 694), in this sense, the quantum statistics work out in both temporal directions—basically, the Born Rule applies equivalently to the past and the future. They analyze three models (a lattice model, the quantum mechanics with universal position localization model for a single particle, and the apparent monotonic increase of energy) in which they show that the underlying dynamics is time-reversal symmetric, and that any asymmetry actually emerges from special boundary conditions ([7], p. 677–678).

The proposal is very attractive and to a good extent follows my intuition about what a time-reversed spontaneous collapse dynamics should look like. However, Bedingham and Maroney’s proposal strongly depends on assumptions (a) and (b), since, as they say: “the wave-function is seen as a convenient way to encode the collapse history and it is this procedure which introduces the time asymmetry. (…) we return to the idea that it is not the diffusion of the wave-function, but instead the location of the collapses that are fundamental” ([7], p. 688). For the proposal to hold, the wave-function should be rendered as unreal. But, even more, the reconstruction of time-reversed, equivalent processes depends on previously knowing the set of collapses in one time direction ([7], p. 686) —for a given set $\left\{{z}_{i}\right\}$ of collapse outcomes, it is possible to obtain *the same* collapses but in the reverse order. The trick is to put diffusion processes aside and just focus on the set of objective collapses as what is fundamental for the theory.

I partially agree with Bedingham and Maroney. Spontaneous collapse theories are indeed TRI. More precisely, they are weakly TRI since they predict the same probabilities for processes in both directions of time via the Born Rule and their stochastic dynamics. Nonetheless, it seems to me that the assumptions (a) and (b) are not necessary. For instance, it is not necessary to assume (b), that is, that the wave-function has an epistemic role. In fact, this assumption can look a bit ad hoc since the epistemic nature of the wave-function is crucial to eliminate some asymmetries in the models that Bedingham and Maroney analyze. Although they explicitly mention that such an assumption is not necessary, it is not clear how the time-reversal symmetry can hold without it. Assumption (a) may, in turn, be too shallow—collapse outcomes are just definitive values for position, but it does not shed light on the ontological aspect of such outcomes. In the philosophical literature on the matter, it is regarded that an appropriate ontology for spontaneous collapse theories is either quantum state monism [38] or a primitive ontology, as an ontology of flashes or of a mass-density field [2, 4, 10, 19]. To assume that collapse outcomes are primitive may lead to a too shallow ontology, requiring further explanation,but it also hampers us to properly evaluate strong TRI. And in order to assess strong TRI, we should go deeper in GRW ontology. In what follows, I will argue that strong TRI is violated in GRW-type theories, in particular, in GRWm.

In the debate about the ontology of quantum mechanics, GRWm bridges the gap between the wave-function, on the one side, and matter, on the other, by proposing a *primitive ontology*. This consists of a mass-density field that occupies the entire physical space, having varying degrees of density in different regions of space. GRWm’s ontology is then one of a gunk [15], a continuous stuff. In such a theory, a variable $m(x,t)$ for every point $x \in {\mathbb{R}}^{3}$ represents such a mass-density field. The connection between the mass-density field and the evolution of the wave function is one of dependence—the latter completely determines the former at any time. In this way, the evolution of the wave-function determines the degrees of matter density in the physical space. For instance, a spontaneous collapse of the wave-function in the configuration space for a single particle will be represented by a high density of matter in the physical space at a point (or at a narrow region). This is represented in terms of the mass-density operator $m(x,t)$.

So, the bone of contention is whether GRWm is strong TRI or not. To assess this, we need to know whether time reversal preserves the ontological content of GRWm as well. Imagine now a many-particle universe. Since GRWm is weakly TRI, quantum superpositions may evolve either with $\psi \left(x,t\right)$ or $T\psi \left(x,t\right)={\psi }^{*}\left(x,-t\right)$. The forward-evolving wave-function for a many-particle system will evolve according to the standard quantum evolution for a while, but it is to undergo a series of stochastic collapses as time goes by (remember that the higher the number of particles, the more frequent the collapses will be). In the physical space, the ontological content of GRWm will be given by the different values that the mass-density operator adopts for different space points at different times—it will show high concentration of matter appearing here and there as spontaneous collapses occur. So, *in the ontology*, the future-headed evolution of the many-particle system will display a specific variation in the distribution of the continuous matter-density field in the physical space during a span of time, $\Delta t$. This variation will be given in terms of differences in the degrees of matter density throughout the physical space. Let us call *f*DM (the *f* stands for ‘forward’, DM for “distribution of matter”) a specific variation of the matter-density field in the forward direction of time.

What happens when the GRWm dynamics is time reversed? We will now have a backward-evolving wave-function in the configuration space, given by ${\psi }^{*}(x, -t)$. This time-reversed wave-function will undergo collapses in the past direction of time by the same statistics and rules as $\psi$. So far, nothing new. But when we look at the ontological content of both evolutions, things will almost certainly look quite different. The backward-evolution of ${\psi }^{*}$ will manifest itself in the ontology as a specific pattern of variations in the distribution of the continuous matter-density field in the physical space during a span of time, $\Delta t$, *towards the past*. This is to bring about a specific distribution of matter density, *b*DM (the *b* stands now for ‘backward’). When we compare *b*DM with *f*DM, some ontological content is displayed according to solutions of the GRW dynamics, that is, some distribution of the concentration of matter in space. But the crucial point is that the ontological contents will almost certainly be different. That is, the distribution of the concentration of matter in space will not be equivalent. In both cases, we will have collapses occurring spontaneously; in both cases, we will obtain a distribution of high-low concentration of matter that varies as time goes by; but, and this is the crucial difference, the collapses are bound to happen differently in $\psi$ and ${\psi }^{*}$. This means that the collapses will almost certainly happen at different times and at different places in both *f*DM and *b*DM, yielding different patterns in the ontologies. I said repeatedly “almost certainly” because there might be a very low probability that they coincide, but that would be an astonishing result (almost a miracle). The reason is simple: there is no dynamical mechanism in GRWm that guarantees that collapses will occur exactly at the same moment and at exactly the same place both in the future and in the past.

It follows from this that GRWm violates strong TRI, since it does not preserve the patterns of the distributions of matter in the forward and in the backward direction of time. By looking at how the distribution of matter density varies, we can substantially distinguish between *f*DM and *b*DM, and thereby, we can substantially distinguish between the future-to-past and the past-to-future direction.^14^ Note that this result is perfectly compatible with GRWm being weakly TRI—what the theory predicts, what the theory says, will be the same in terms of probabilities; but the content of what becomes actual (which reflects itself in the variations of the matter-density field) is to be different. Therefore, the ontological content is not preserved.

This is good news for temporal primitivism–if GRWm violates strong TRI, then GRWm can well serve to ground temporal primitivism in the quantum regime. However, two arguments could downplay this view.^15^ First, it may be argued that there is nothing specially quantum in the argument since classical stochastic systems would manifest a similar asymmetry. In this sense, an analogous argument would be also valid for such classical systems, downplaying the role of GRWm in introducing a primitive direction of time. After all, the nature of the temporal asymmetry would boil down to stochasticity, both in the classical and quantum regime. To see it clearly, consider that many classical systems evolve stochastically in the forward direction of time (e.g., Brownian particles). They would display a certain pattern in their statistical behavior as time goes by. If you now prepare the Brownian particles but make them evolve backward in time, they will almost certainly display a different pattern *in the past*. If this is so, then it is possible to distinguish between both directions of time because the patterns of the Brownian particle behavior are bound to be different. Therefore, there is nothing specially quantum in the sort of temporal asymmetry that GRWm brings about—such an asymmetry ultimately relies on the stochastic nature of some physical systems, both classical and quantum.

It is true that there seems to be a prima facie analogy between the quantum and the classical case. Yet, I believe that there are two key differences. To begin, in GRW, it is the fundamental dynamical equation (Eq. 4) that is stochastic, whereas in the classical case the stochasticity is typically taken as epistemic, arising from the lack of information about the exact initial conditions of each classical system. The fundamental classical dynamics is still deterministic, according to the classical dynamical laws (e.g., Newton’s laws). So, the stochastic behavior in the quantum case is “built in” the dynamics, whereas in the classical case depends on our ignorance about the initial states of all systems, over a deterministic fundamental dynamics. Second, in GRW, the dynamics describes the behavior of collapses in the configuration space, which translates into variations in the value of the matter-density field in the space and time. So, in some sense, the GRWm dynamics “brings about” the basic ontology. This is probably easier to see in GRWf, where flashes are the primitive ontology of the theory. The stochastic production of flashes “brings about” the ontology of the theory, not merely a description of the behavior of pre-existing events—without collapses, there is nothing in space and time. In the classical case, the existence of the fundamental ontology does not depend in any relevant sense on the dynamics. Thus, the fact that GRW is strongly non-TRI means that the stochasticity is built in the fundamental dynamics and that the basic ontology itself changes when time is reversed. In the classical case, the temporal asymmetry seems to be matter of the lack of information about the initial states of the classical systems and independent of the ontological content of the theory. Therefore, the analogy is interesting, but a complete parallel cannot be made.

The second objection runs as follows. In the case where *f*DM and *b*DM are obtained, there is a temporal asymmetry on which temporal primitivism can be based. But, it can be argued, there is nothing special about time, since the same initial state can be prepared twice (say, $\psi \left(x,{t}_{0}\right)$ and $\psi {^{\prime}}\left(x,{t}_{0}\right)$), make them evolve forward in time, displaying different distributions of matter (say, *f*DM and *f*DM’). Therefore, the temporal asymmetry between *f*DM and *b*DM is analogous to the non-temporal asymmetry between *f*DM and *f*DM’. In this case, it is clear that the nature of both asymmetries is equally built in the dynamics, so no difference in that aspect. This is an interesting case to delve into, though I do not have enough space to do it properly here. Let me say just a few words. In some respect, the temporal asymmetry between *f*DM and *b*DM is a special case of an asymmetry for any distribution of matter based on a fundamental stochastic dynamics (as GRW). It is thus true that the reversal of the direction of time is not special in this respect. However, temporal primitivism can still be held—the argument does not show that *f*DM and *b*DM are not fundamentally asymmetric (which would be a case against temporal primitivism), but that *f*DM and *b*DM is a special case. It follows that a fundamental asymmetry is produced when time is reversed (in which temporal primitivism is grounded), *and* also in other cases where the reversal of time is not involved. The argument makes temporal primitivism less special, but not false. The argument is really interesting since it shows that the nature of temporal primitivism in the GRW case is dependent on the stochastic nature of a dynamics like GRW.^16^

## Conclusions

In Sect. 2, I rephrased the problem of whether the natural world comes equipped with a direction of time direction in terms of whether it possesses a B-structure. Temporal Primitivism, in its dynamical version, is one of the most promising ways to know it by drawing the attention to the concept of TRI. Non-primitivists readily point to the fact that it seems that our most fundamental physical theories are TRI. This, of course, runs for quantum theories as well. In the non-relativistic regime, it is the TRI of the Schrödinger equation which renders the quantum world as lacking a fundamental directionality of time. According to the anti-Primitivist Argument, then, temporal primitivism is unwarranted in non-relativistic quantum mechanics.

My strategy to resist the argument was to rely on GRWm and show in which sense it can be said to violate strong TRI. First of all, non-relativistic quantum mechanics suffers from dynamical and metaphysical underdetermination. To ask whether standard quantum mechanics is TRI is not quite precise; we should rather ask whether a specific quantum theory is TRI. If there is an empirically adequate quantum theory that allows her to circumvent the TRI-based argument, she could pick it out as a physical basis for temporal primitivism. The only constraint is that the theory violates TRI in a relevant sense.

A promising candidate for this is the spontaneous collapse theories, in particular, GRWm. But, in which sense can it violate TRI? In Sect. 2.2. I argued that TRI can be said in two ways—strong and weak. Whereas weak TRI centres in preserving the formal relations among elements within a mathematical structure (preserving its space of solutions), strong TRI takes also into account the preservation of the ontological content. The primitivist counterargument may then go on as follows. By focusing on MQ3 in the anti-Primitivist Argument, it can be said that there is a quantum theory (GRWm) that violates strong TRI since the ontological content of the theory changes under time reversal. The fact that GRWm is weakly TRI brings about two evolutions for wave functions, that are ontologically represented as two patterns of density distribution of the matter field in the physical space (*f*DM and *b*DM). The fact that GRWm violates strong TRI renders them inequivalent.

This is enough to equip the world with a B-structure and to uphold temporal primitivism in its dynamical version. To begin, nothing in the matter-density field ontology itself introduces a temporal asymmetry (since without the dynamics the ontology is static). If only the dynamics is taken into account, we may miss how and when the temporal asymmetry comes about (since the dynamics only minds structural aspects). However, when both elements are taken into consideration, we can more clearly see the temporal asymmetry that is built in to the theory: the distributions of the matter density field in the future (*f*DM) and in the past (*b*DM) are bound to be different, inequivalent. Or, to put it differently, the very distribution of the matter-density field already embodies a temporal direction, because if time’s direction were different, the matter-density distribution would be also different So, we can substantially distinguish between past-headed and future-headed GRWm evolutions. From this, we can give the world a B-structure *if* GRWm is adopted. Such a B-structure cannot be reduced to (or emerge from) any directionless C-structure. Thus, if the existence of alternative quantum theories is accepted; if the distinction between strong and weak TRI is adopted, GRWm can well be a strong case in favour of temporal primitivism because it violates strong TRI.

## Data Availability

Not applicable.

## Footnotes

1 Carlo Rovelli [57] argues for a radical relational view of time where it does not even have a C-structure. In the covariant formulation of loop quantum gravity time relabeling symmetry holds, which entails that time lacks an order. For the purposes of this paper, I will assume that any theory of time must have at least a C-structure, putting theories of quantum gravity aside. This won’t affect my argument, since I’m more concerned with the C- and B-structures distinction. For discussion on this, see Gryb and Thébault [23].

2 A good argument for supporting this premise could run as follows. Desirably, dynamical symmetries of a theory should match its space–time symmetries (see [14], p. 46, [41], p. 202). That is, symmetries found at the level of the dynamics of a theory are symmetries of the space–time structure that supports it. If time’s direction is to be found in the underlying space–time structure, a straightforward way to know it is by looking into the symmetries of the dynamics.

3 Belot mentions two clear cases –the harmonic oscillator and linear homogeneous partial differential equations (such as the heat equation, wave equation, etc.). As Belot contends “under any ordinary reading, they admit solutions that represent situations in which nothing is happening and others that represent situations in which plenty is going on” (p. 325).

4 Clearly, this observational content is to be assessed counterfactually –were time reversed, the observational content would be different. However, this is not easy to assess even in counterfactual scenarios –since we cannot perform experiments in time-reversed situations, how can we even come to know what the time-reversed observational content would be like?

5 Other alternatives refer to modal or physical equivalence [54] or to objectivity [41]. I will not discuss which view is the most adequate to construe a conceptually substantial notion of symmetry in general, and of TRI in particular.

6 Whether alternative quantum theories are completely empirically equivalent is a problem in itself. On the one hand, all quantum theories equivalently and successfully account for the quantum statistics obtained by standard quantum mechanics. On the other, some quantum theories do make different predictions —Bohmian Mechanics and spontaneous collapse theories make predictions that diverge from standard quantum mechanics. In any case, as it is common in the literature, I will assume that all alternative quantum theories are *at present* largely empirically adequate and, in that sense, suffer from underdetermination by evidence.

7 To clarify this point, let me give an example. De Broglie-Bohm’s pilot wave theory shares the same dynamics as Modern Bohmian Mechanics (as formulated by Dürr, Goldstein and Zangui in the 90 s, though there are minor differences). However, they bear different ontologies –the former adopts a dualist ontology of particle and fields, whereas the latter does not. There is, therefore, metaphysical underdetermination, but not dynamical underdetermination.

8 This manoeuvre could look a bit ad hoc, but it is not so. Provided that quantum theories suffer from underdetermination by the evidence, we cannot pick any of the theories by their empirical success or logical coherence only, but by relying on other super-empirical or metaphysical virtues (see Callender 2020). When empirical adequacy cannot settle a rivalry among alternative theories, it ought to be solved by other means. Though in naturalized metaphysics the reasoning is typically one that tailors one’s metaphysics conforming with science, metaphysical underdetermination gives room for doing just the inverse –the physics may be accommodated to meet one’s metaphysical view –after all, empirical adequacy is out of discussion.

9 The first formulation of a robust spontaneous collapse theory is the *Quantum Mechanics with Spontaneous Localization* (QMSL), primarily proposed by Ghirardi, Rimini and Weber in 1986 (see [21]. After facing some criticisms, a new collapse theory was thus introduced few years later by Pearle [42] and Ghirardi, Pearle and Rimini [20]. This new theory, usually referred to as *Continuous Spontaneous Localization* (CSL), replaces the discontinuous jumps of QMSL by a continuous stochastic evolution in the Hilbert space, also called ‘a diffusion process’. Though both theories are different, for my purposes here the details are not relevant, so I will keep referring to them as GRW in general with some elements of CSL.

10 The frequency of a collapse for a single, isolated particle is of the order of ${10}^{-16}{s}^{-1}$

11 The width $\sigma$ of the multiplying Gaussian curve is of the order of ${10}^{-7}$ *m*

12 Technically, equation (Eq. 4) is the dynamical equation for the CSL model.

13 It may be suggested that orthodox quantum mechanics and measurement-induced collapses are enough to prove quantum mechanics to violate even weak TRI. Why then bother with GRW? First, orthodox quantum mechanics has vastly been criticized in the philosophical literature as an unattainable quantum theory. Second, it has been argued that it does not allow any straightforward implementation of time reversal (see [32]. Within the family of collapse theories, GRW models (and similar) have a better reputation than orthodox quantum mechanics.

14 To be more precise, we cannot know which one is the “future-directed” and the” past-directed” evolution. But this is not the point –labels ‘past’ and ‘future’ are conventional. What matters is to find a way to distinguish them in the physics. Since *f*DM and *b*DM bear different ontological content (a different pattern in the distribution of density in the matter field), there is a way to distinguish them.

15 I thank an anonymous reviewer for drawing the attention to these two objections.

16 I thank two anonymous reviewer for suggesting this criticisms
